# Biasing AlphaFold2 to predict GPCRs and kinases with user-defined functional or structural properties

**DOI:** 10.3389/fmolb.2023.1121962

**Published:** 2023-02-16

**Authors:** Davide Sala, Peter W. Hildebrand, Jens Meiler

**Affiliations:** ^1^ Institute of Drug Discovery, Faculty of Medicine, University of Leipzig, Leipzig, Germany; ^2^ Institute of Medical Physics and Biophysics, Faculty of Medicine, University of Leipzig, Leipzig, Germany; ^3^ Center for Structural Biology, Vanderbilt University, Nashville, TN, United States; ^4^ Department of Chemistry, Vanderbilt University, Nashville, TN, United States

**Keywords:** AlphaFold, GPCRs (G-protein-coupled receptors), kinases, structure prediction, protein function

## Abstract

Determining the three-dimensional structure of proteins in their native functional states has been a longstanding challenge in structural biology. While integrative structural biology has been the most effective way to get a high-accuracy structure of different conformations and mechanistic insights for larger proteins, advances in deep machine-learning algorithms have paved the way to fully computational predictions. In this field, AlphaFold2 (AF2) pioneered *ab initio* high-accuracy single-chain modeling. Since then, different customizations have expanded the number of conformational states accessible through AF2. Here, we further expanded AF2 with the aim of enriching an ensemble of models with user-defined functional or structural features. We tackled two common protein families for drug discovery, G-protein-coupled receptors (GPCRs) and kinases. Our approach automatically identifies the best templates satisfying the specified features and combines those with genetic information. We also introduced the possibility of shuffling the selected templates to expand the space of solutions. In our benchmark, models showed the intended bias and great accuracy. Our protocol can thus be exploited for modeling user-defined conformational states in an automatic fashion.

## Introduction

X-ray crystallography and cryogenic electron microscopy (cryo-EM) are two widely used techniques for determining the detailed structures of biomolecules at the atomic level (Vénien-Bryan et al., 2017; Wang and Wang, 2017). For structure-based drug discovery and design, having at least one high-accuracy structure is essential (Congreve et al., 2020). Despite recent advances in technology have made more protein structures available (Callaway, 2020), their experimental determination is still a difficult and costly process with a high risk of failure (Lyumkis, 2019). In fact, experimental protein structures represent only a small fraction of the complete set of known protein sequences (The Uniprot Consortium, 2019; Burley et al., 2021). Furthermore, one structure only represents a snapshot of a certain protein state, and may not necessarily be sufficient to understand the overall mechanism of operation. This limitation has important implications for drug discovery, especially for common drug targets such as G-protein-coupled receptors (GPCRs) and kinases, which are known to modulate cellular behavior by switching among multiple structurally different functional states (Attwood et al., 2021; Yang et al., 2021).

The 14th edition of Critical Assessment of protein Structure Prediction (CASP14) has recognized AlphaFold2 (AF2) for its impressive accuracy in predicting monomeric protein structures *de novo* (Jumper et al., 2021). AF2 makes it straightforward to predict a protein structure from a protein sequence and has provided millions of protein models with estimated accuracy (Tunyasuvunakool et al., 2021). Since the emergence of AF2, a number of deep learning-based methods have been developed with the same goal of predicting protein structures at experimental accuracy (AlQuraishi, 2021; Baek et al., 2021; Chowdhury et al., 2022; Lin et al., 2022). Among them, RoseTTAFold was the first approach that was able to predict both active and inactive GPCR conformations by using templates in a uniform functional state, outperforming comparative homology modeling methods (Baek et al., 2021). This achievement has sparked interest in developing workflows to predict multiple native conformations of a protein target with the state-of-the-art AF2 implementation.

To date, a number of AF2 customizations that adopted different concepts are available (Del Alamo et al., 2022; Heo and Feig, 2022; Stein and Mchaourab, 2022; Wayment-Steele et al., 2022). Del Alamo and co-authors took advantage of a shallow multiple sequence alignment (sMSA) to collect an ensemble of structures, among which multiple native conformations of GPCRs and transporters were identified (Del Alamo et al., 2022). Alternatively, SPEACH_AF (hereafter SPEACH) masked multiple positions in the multiple sequence alignment (MSA) to switch the prediction toward alternative conformational states that were less represented in the MSA (Stein and Mchaourab, 2022). Another protocol removed the MSA (noMSA) and prepared a local database of state-annotated GPCRs to perform AF2 template-based modeling (Heo and Feig, 2022). These methods for sampling conformational changes in proteins have shown great potential, but also have some limitations, such as a reduced breadth of sampled conformations or a high dependence on the structural features of selected templates.

Here, we update our previous protocol (sMSA) to facilitate the collection of templates with user-defined functional or structural properties of GPCRs and kinases. Templates are automatically filtered and retrieved from an annotated database in accord with the specified functional or structural criteria. Through a calibrated balancing of genetic and template-based features, our protocol samples equal or better active GPCR states than all the peer-reviewed methods for sampling alternative states. On a difficult target, randomizing templates to explore the available structural space significantly improved accuracy. In modeling kinase conformations, our protocol enriched the predicted ensemble with models carrying user-defined structural features.

## Methods

We updated our previous modified ColabFold version (Del Alamo et al., 2022; Mirdita et al., 2022) and our python interface to allow users to specify functional or structural properties of templates for modeling GPCRs and kinases. The new implementation and accompanying documentation can be found at https://github.com/meilerlab/AF2_GPCR_Kinase.

### GPCRs benchmark

Target PDBs for Lutropin-choriogonadotropic hormone receptor (LSHR), Melatonin receptor type 1A (MTR1A), Prostaglandin E2 receptor EP4 subtype (PE2R4), Beta-1 adrenergic receptor (ADRB1), Parathyroid hormone/parathyroid hormone-related peptide receptor (PTH1R) and Frizzled-7 (FZD7) were 7FII, 7VGY, 7D7M, 7JJO, 6NBF and 6WW2 respectively (Su et al., 2020; Duan et al., 2021; Nojima et al., 2021; Wang et al., 2022). The protein regions corresponding to transmembrane helices (TM-RMSD) were retrieved from GPCRdb (Kooistra et al., 2021). Four workflows were evaluated to predict the active state of GPCRs: ActTemp+sMSA was run with eight sequence clusters and 16 extra cluster sequences combined with the automatic detection of “Active” templates not belonging to the same subfamily. Those number of sequences were chosen to provide evolution-based structural information without changing the activation state inferred from templates. In particular, the script takes the AF2 generated list of templates ranked by sequence identity and filters out all the PDBs not matching the user-defined activation state in accord to GPCRdb annotation. Here, the top 4 templates were used. For LSHR, MTR1A, PE2R4, ADRB1, PTH1R and FZD7 those were (sequence identity in parenthesis): 6H7L_A (20.6%)-6IBL_A(15.9%)-6K41_R(23.1%)-6K42_R(23.7%), 6H7L_A(26.6%)-7P00_R(23.7%)-6IBL_A(19.9%)-7RMG_R(22.7%), 7E32_R(21.9%)-7CKY_R(20.4%)-7CKW_R(19.2%)-7JVP_R(20.4%), 6MXT_A(37.1%)-7CKY_R(36.8%)-7CKW_R(36.8%)-7JVP_R(37.4%), 7F16_R(35.8%)-6M1I_A(26.0%)-6P9Y_R(30.5%)-6VN7_R(32.0%) and 6XBM_R(25.7%)-6XBK_R(19.0%)-6OT0_R(27.2%)-7D76_R(18.3%) respectively. Other AF2 parameters were kept as in our previous pipeline - named sMSA - that used 16 sequence clusters and 32 extra cluster sequences without any template and no recycling (Del Alamo et al., 2022). To remove the MSA (noMSA run), the same implementation published previously was adopted (Heo and Feig, 2022). These runs were then carried out using the GPCRdb API (Application Programming Interface) rather than a local GPCR database to avoid mismatches between the pool of available templates. The SPEACH protocol was applied with a sliding window of 10 masked residues (Stein and Mchaourab, 2022). Thus, the number of models collected with SPEACH was higher than the 50 models collected with other protocols. Unfolded models were discharged.

To assess the impact of randomizing templates, the inactive state structure of Leukotriene B4 receptor 1 (LT4R1, PDB 7K15) was used as a target (Michaelian et al., 2021). The MSA for the aligned regions was removed, and 50 models were generated with and without randomizing templates. The templates used for the models without randomization were 6VI4_A(27.5%)-4ZUD_A(20.0%)-4YAY_A(20.1%)-4N6H_A(20.2%).

### EIF2AK4 kinase benchmark

All the experimental structures available were absent from the AF2 training set. Models were predicted by using exactly the same ActTemp+sMSA protocol adopted for GPCRs predictions but with 20 templates instead of 4. The DFG, aC_helix, and Salt bridge K^III.17^ and E^αC.24^ structural features as well as the activation loop orientation used to collect templates were defined according to the KLIF database (Kanev et al., 2021). Unfolded models were discarded.

## Results

**SCHEME 1:**
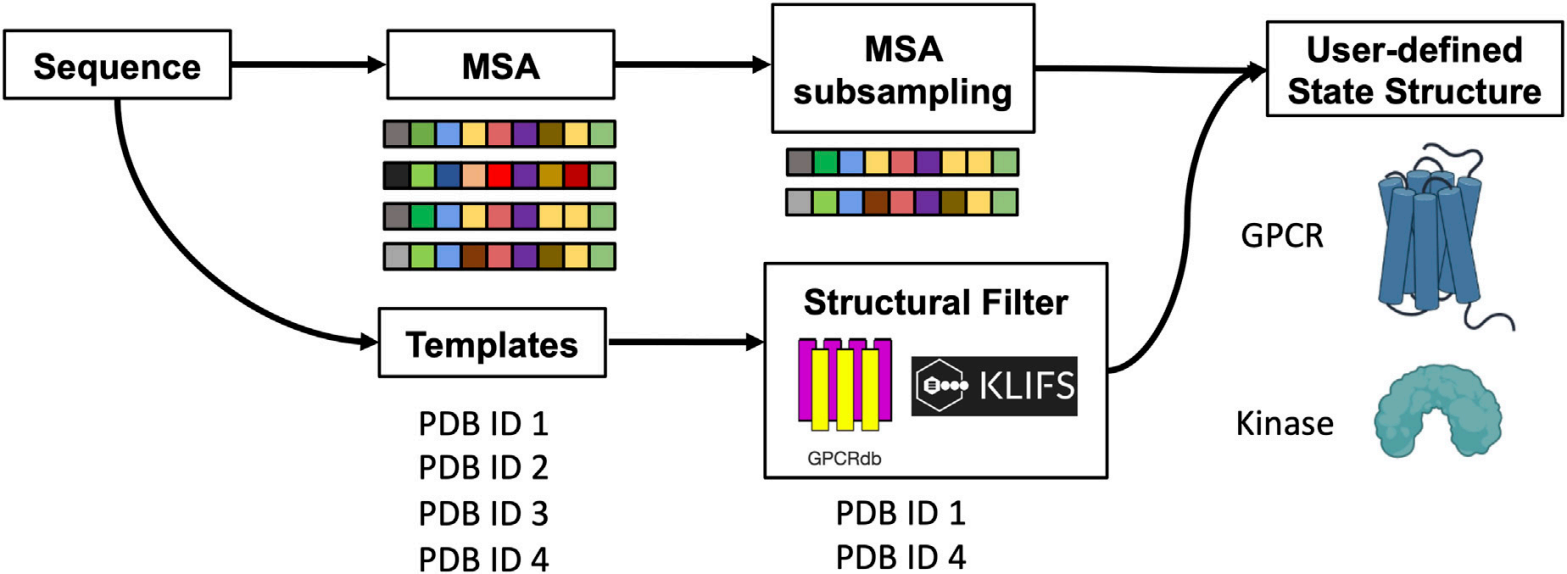
Schematic representation of the method. The protein sequence is used to collect MSA and templates. A subset of sequences and templates are collected by randomly subsampling the MSA and by interrogating webservers to filter templates with user-defined structural properties. The predicted ensemble of structures is biased toward the intended conformation.

The original pipeline that was developed to sample alternative conformations was expanded to improve the prediction of GPCRs and kinases in a specific conformational state. Here, templates are selected through structural filters and the resulting structures are combined with genetic information coming from a subset of the MSA to predict models carrying the desired structural properties at high accuracy (Scheme 1). In particular, users can now specify the activation state of GPCRs and the script will look for templates that match that state or are bound to a signaling protein. To do so, one of the following labels must be declared: “Active”, “Inactive”, “Intermediate”, “G protein”, “Arrestin”. For kinases, users can select specific structural feature values and the script will search for templates that match those criteria. Allowed values for the corresponding structural feature are 1) DFG: “out”, “in”, “out-like”, “all”; 2) aC_helix: “out’, “in”, “all”; 3) Salt bridge K^III.17^ E^αC.24^: “yes”, “no”, “all” (McClendon et al., 2014). Optionally, the list of templates that pass the sequence and structural filters can be randomized to explore the available structural space.

In the sections below, we demonstrate how selecting templates in accord with functional or structural properties and combining those with genetic information can influence the predicted structural features of the models. We also show the results of randomizing templates on a difficult target.

### Combining a shallow MSA with state-annotated templates achieves state-of-the-art accuracy in predicting GPCRs active state

**FIGURE 1:**
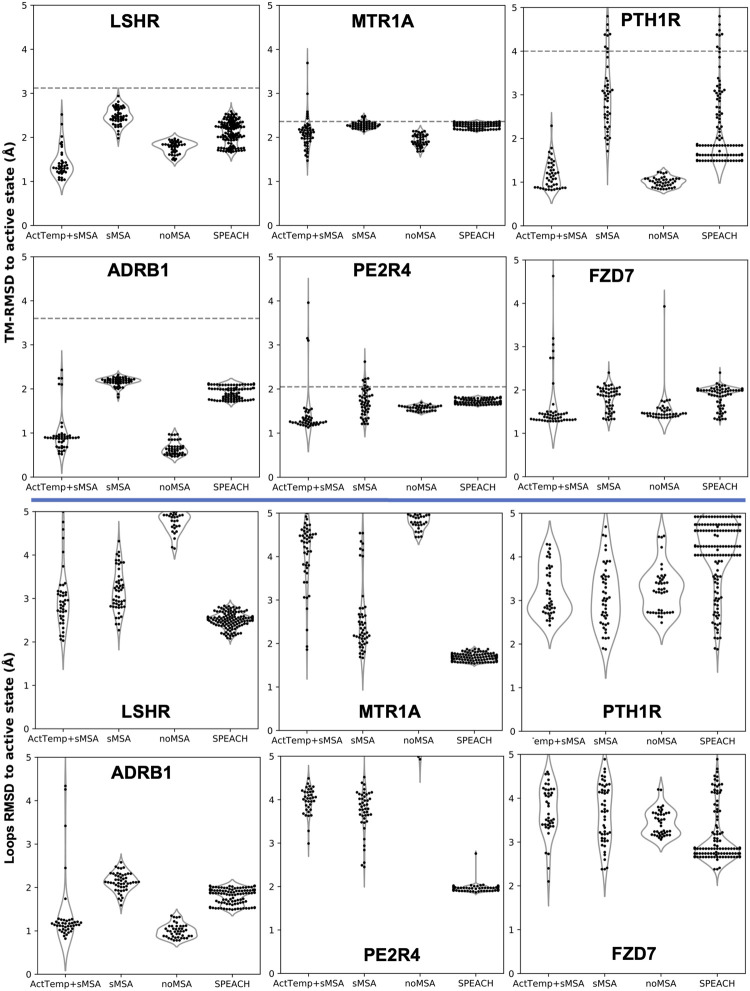
AF2 accuracy in predicting active state GPCRs with different protocols. ActTemp+sMSA was predicted with templates in the active state and a shallow MSA, sMSA with a shallow MSA only, noMSA without a MSA for templates aligned regions, SPEACH with a sliding window masked MSA. TM-RMSD between experimental active and inactive structures is shown as a dashed line.

Our new pipeline was used to predict GPCR models by combining a very shallow MSA with the automatic detection of the best 4 active templates from GPCRdb (ActTemp+sMSA). The benchmark set of these GPCRs consisted of six proteins: LSHR, MTR1A, PE2R4, PTH1R, FZD7 and ADRB1. The first three class A receptors were predicted with the lowest accuracy in a broad benchmark in which the active state was modeled without MSA (Heo and Feig, 2022). PTH1R and FZD7 are members of class B and class F family, respectively. Instead, the active state of ADRB1 was included because the inactive state was part of the neural networks training set. Thus, we targeted the active state with the specific aim of assessing the ability of our implementation to overcome the neural networks preference for the inactive state. For each method, we measured the accuracy as Cα-RMSD (root-mean-square deviation) of the transmembrane helices (TM-RMSD) as well as of the loops with respect to the experimentally determined structure. Our implementation was compared to AF2 workflows designed to sample alternative protein conformations. ActTemp+sMSA consistently generated models with near or subangstrom accuracy for all the GPCRs TM helices, showing state-of-the-art accuracy (Figure 1). Interestingly, our approach and noMSA were the only methods able to overcome the ADRB1 inactive state bias and accurately model the active state with an average accuracy of 0.5 Å on TM helices and 1 Å on loops. On the remaining targets, loops were in general better modeled by protocol leveraging on genetic information than those on templates. In particular, SPEACH—that does not reduce the MSA depth—has shown a consistent good accuracy. By comparing the two methods that leverage on templates (ActTemp+sMSA and noMSA), loops were on average better modeled by the former probably due to the contribution of genetic information compensating for missing or poorly conserved loops in the selected templates.

**FIGURE 2:**
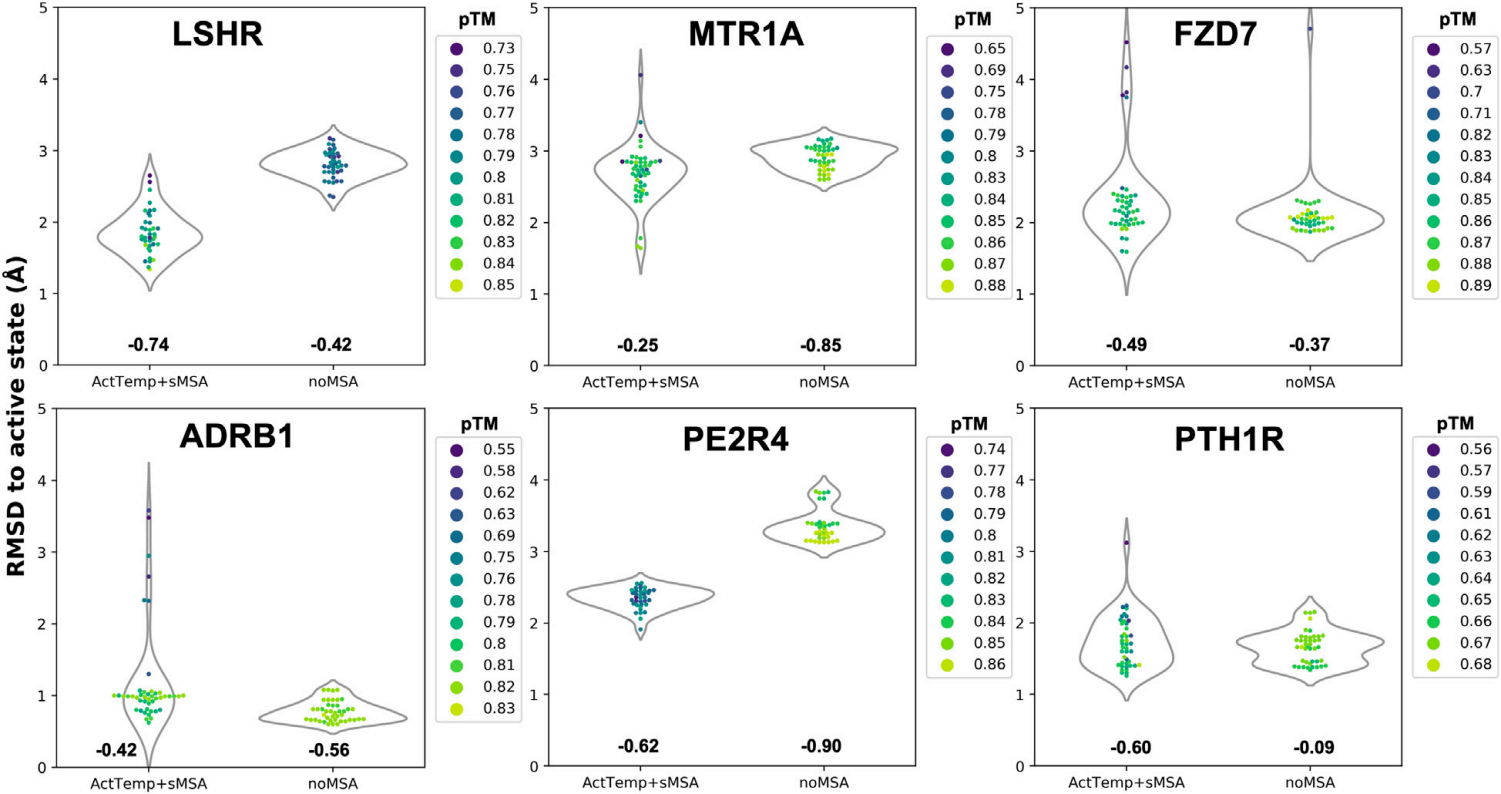
Correlation between pTM and global RMSD per target. Spearman correlation for each ensemble is indicated below each violin plot.

Given the separated evaluation of TM helices and loops accuracy, we measured the pTM score per model and assessed Spearman correlation between pTM and global RMSD for each ensemble (Figure 2). Overall, ActTemp+sMSA generated equally or better active state models than noMSA mainly due to higher accuracy in loops modeling. Within each ensemble, correlation is often reasonable and more importantly the best models are often assigned with the highest pTM scores with very few exceptions. However, pTM scores between the two protocols do not seem correlating well with accuracy. In other words, pTM scores often cannot correctly discriminate which protocol generated best active structures.

### Shuffling templates in a homogenous functional state can improve accuracy

**FIGURE 3:**
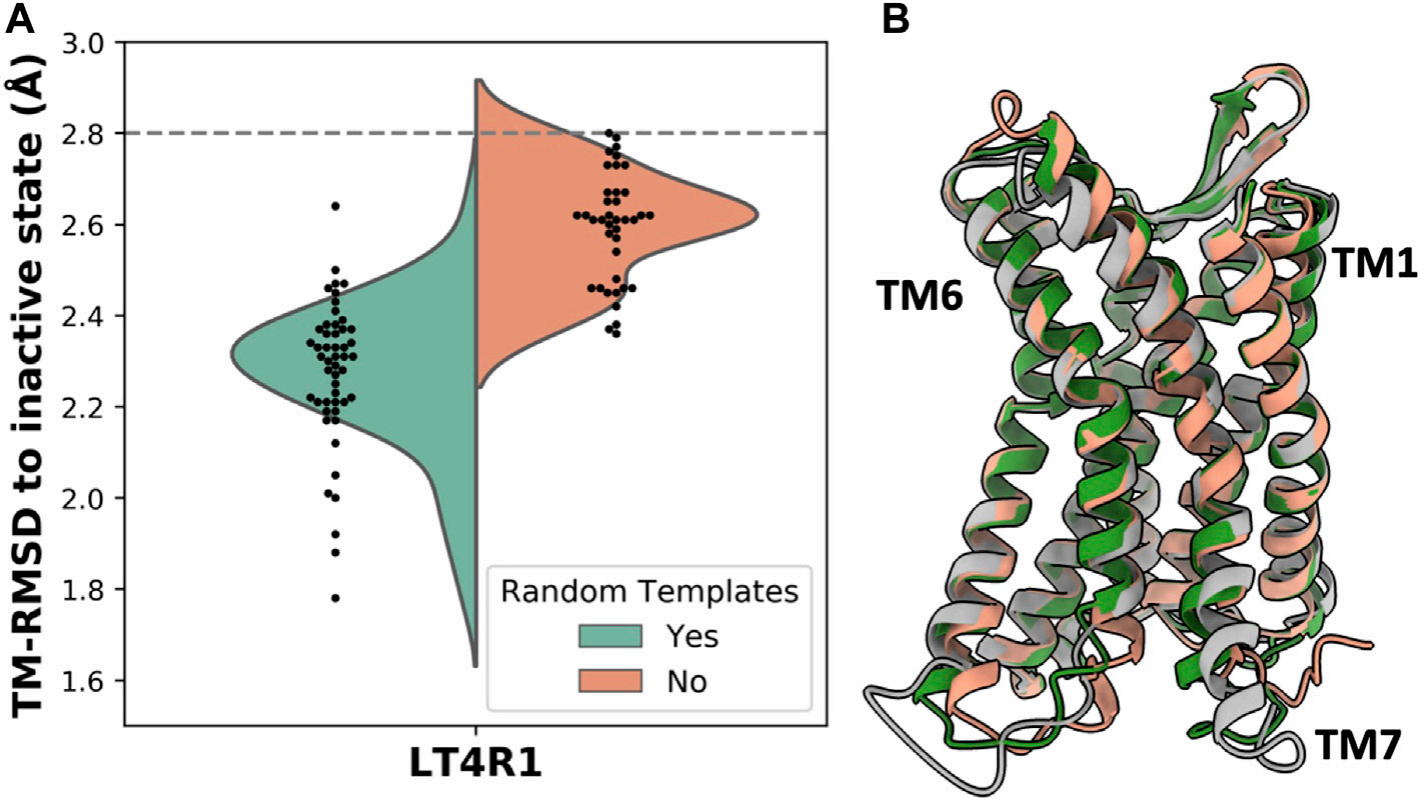
Accuracy in predicting the LT4R1 inactive state with and without randomizing templates. **(A)** TM-RMSD distribution of models. TM-RMSD between experimental active and inactive structures is shown as a dashed line. **(B)** Superposition of the best model from the random templates ensemble (green) and without randomizing templates (orange) to the experimental structure (gray).

Given that subsampling the sequence space (i.e., the MSA) returns different models, we hypothesized that randomly selecting a subset of templates can potentially yield more accurate models. To test this, we removed the genetic information within the AF2 pipeline and generated 50 models with and without randomizing inactive templates. For each model, our script selected 4 random inactive state structures from GPCRdb that passed the sequence similarity filter. Accuracy was measured as TM-RMSD from the inactive state structure of LT4R1 (PDB 7K15). The exploration of the structural space defined by the ensemble of all the inactive templates resulted in more accurate models compared to using the top 4 templates (Figure 3A).

The superposition of the best model in the two ensembles shows improved fitting of the long TM7 helix and better modeling of TM1 and TM6 when using random templates (Figure 3B).

### User-defined structural features to bias kinase modeling

**FIGURE 4:**
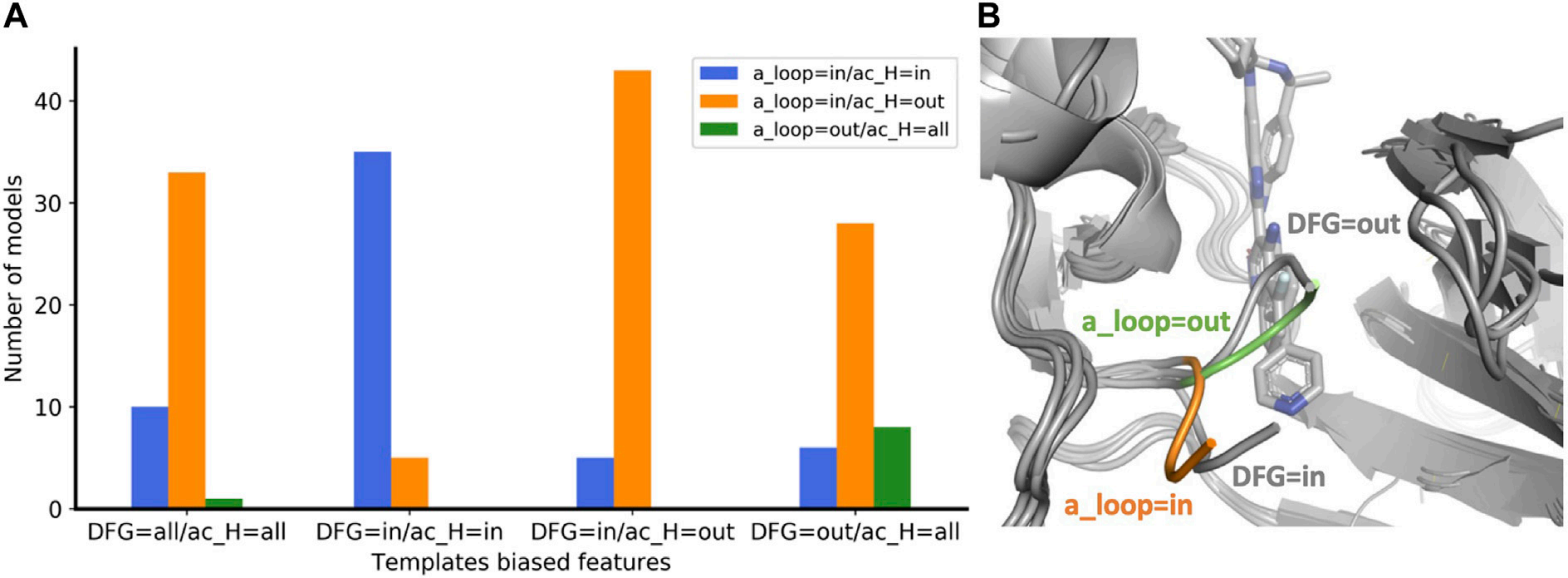
**(A)** Enrichment of eif2k4 kinase models with structural properties corresponding to the biased template features used. The four ensembles were calculated with a different “DFG/ac_H” templates bias. For each ensemble, the number of models with the three “a_loop/ac_H” conformational feature combinations are shown with a different color bar. **(B)** Superposition of two models with a_loop=in and a_loop=out to the two corresponding “DFG=in” and “DFG=out” experimental structures. DFG residues of models with “out” and “in” orientations are shown in green and orange, respectively. Experimental structures of eif2k4 are shown in gray.

The concept of allowing users to define structural features of GPCR templates was also applied to kinases using the KLIF webserver (Kanev et al., 2021)**.** We implemented the possibility to choose templates differing on three conformational properties: DFG, αC-helix (ac_H), and salt bridge K^III.17^E^αC.24^. The script automatically selects and retrieves templates satisfying user-defined values for these three structural criteria. We assessed the effect on the predicted conformations by modeling the EIF2AK4 (GCN2) kinase. We generated four ensembles of 50 models each with the following templates biased features: 1) “DFG=all/ac_H=all”, i.e. all templates are allowed; 2) “DFG=in/ac_H=in” and 3) ‘DFG=in/ac_H=out’ which differ in the αC-helix position regardless of its rotation, i.e. templates have DFG=in but differ in the ac_H conformation; 4) “DFG=out/ac_H=all”, all the selected templates have DFG=out but ac_H is allowed in any conformation. Because DFG is a multi-criteria parameter, instead of measuring whether the predicted DFG corresponds to the selected DFG templates bias, we evaluated the activation loop (a_loop) position which is well-defined and mostly corresponds to DFG. Without biasing the prediction (DFG=all/ac_H=all), most of the models were found in the “a_loop=in/ac_H=out” conformation, while 20% of the pool was in the “a_loop=in/ac_H=in” conformation, and only one model was found with “a_loop=out” (Figure 4A). By biasing the prediction through the selection of ac_H=in and ac_H=out templates in two different ensembles (DFG=in/ac_H=in and DFG=in/ac_H=out), AF2 generated most of the models in agreement with the templates ac_H position. Accordingly, “DFG=in” templates generated only “a_loop=in” conformations (blue and orange bars) while in the only “DFG=out” ensemble we found a significant number of models carrying the “a_loop=out” conformation (green bar). The superimposition of “a_loop=out” and “a_loop=in” models onto the corresponding experimental “DFG=out” (PDB 7QWK) and “DFG=in” structures (PDB 7QQ6) shows an excellent fitting of DFG loops, with a small discrepancy for ‘DFG/a_loop=out’ likely due to the presence of the inhibitor in the experimental structure (Figure 4B) (Maia de Oliveira et al., 2020).

## Discussion

The prediction of user-defined conformational states of proteins has been a challenge even after the advent of AF2. Previous workflows attempting to solve this problem either do not explicitly predict user-defined structural properties or require the creation of state-annotated local structure databases (Del Alamo et al., 2022; Heo and Feig, 2022; Stein and Mchaourab, 2022; Wayment-Steele et al., 2022). In this work, we propose a pipeline that biases AF2 predictions toward the intended functional state of GPCRs or specific structural properties of kinases. One key aspect of our method is its simplicity in use. By leveraging on the API (Application Programming Interface) of two popular web servers, GPCRdb and KLIFS (Kanev et al., 2021; Kooistra et al., 2021), our script filters templates according to pre-defined structural or functional parameters, allowing for a fully automatic selection of templates without the need for manual inspection or for downloading and updating of databases.

Our results in predicting the active structures of several challenging GPCRs show that combining a shallow multiple sequence alignment (MSA) with templates in a user-defined activation state (i.e. structure annotated as Active, Inactive or Intermediate) outperforms existing AF2 workflows. A direct comparison with models predicted without an MSA (noMSA) suggests that the balanced combination of genetic (MSA) and structural (templates) features may be crucial for achieving high accuracy, especially on loops that are usually less conserved and feature higher structural variance. This balanced mixture enables structural refinement of the desired conformational state while avoiding the overwhelming effect coming from a deep MSA, as previously reported (Del Alamo et al., 2022). Another advantage of a balanced mixture of genetic and structural information is its reduced sensitivity to neural network biases, i.e. the conformational preference of the neural network. In our benchmark, target conformations were four class A and one class B1 GPCRs for which inactive structures were more prevalent than active ones in the AF2 training set. Furthermore, the inactive structure of ADRB1 was directly part of the AF2 training set, thus representing a very strong bias. Indeed, protocols relying solely on genetic information (sMSA and SPEACH) were on average less accurate and completely missed the target conformation for ADRB1. On the other side, ActTemp + sMSA and noMSA depend on the presence of high-accuracy templates. Indeed, ADRB1 was predicted with an astonishing low RMSD value due to the high accuracy of the active state templates on both TM helices and loops.

Shuffling templates to predict the inactive state structure of LT4R1 generated better models than by taking the top four sequence identity templates in the inactive state. Regions that were better modeled were indeed different in the top four templates. Suggesting that despite a lower sequence identity, templates randomly chosen from the remaining pool of inactive state structures may have been more suitable to model this conformational state. This kind of approach can be used to expand sampling without changing the desired structural features, like the activation state of a GPCR.

Our efforts to bias the prediction of a kinase toward user-defined structural properties exploited two important structural components that define its activation state: DFG and αC-helix. While the latter was easier to direct toward the intended position, the former was more difficult likely due to the neural network bias in the training set composition. Despite this, we successfully generated multiple models with “DFG=out” conformation. Given that “DFG=out” structures are needed for structure-based drug design and discovery of type-II inhibitors (Ung and Schlessinger, 2015), our script is well positioned to generate models carrying this crucial structural feature. Frequency of sampling the desired structural features may change protein by protein due to multiple factors such as neural network biases, templates features and MSA composition.

Our work expands the portfolio of AlphaFold2 customizations developed with the aim of predicting multiple conformational states of proteins. Our python interface facilitates the prediction of intended functional or structural properties of GPCRs and kinases and can be further extended to include more properties as needed. We also emphasize the importance that structure- and function-annotated databases had for this work. The expansion of existing databases to include additional annotations and the development of new protein family-based databases would improve or enable automatic calibrated modeling, respectively. This is particularly relevant for receptors and transporters that are known to span multiple conformations in their functional cycle. Together, curated databases and machine learning offer a powerful combination for high throughput modeling at high accuracy and, ultimately, for structure-based drug discovery (Sala et al., 2022).

## Data Availability

Models generated with the described protocol are made available at https://doi.org/10.5281/zenodo.7602488. The python script and corresponding documentation can be found at https://github.com/meilerlab/AF2_GPCR_Kinase.
